# Tissue maintenance of CMV-specific inflationary memory T cells by IL-15

**DOI:** 10.1371/journal.ppat.1006993

**Published:** 2018-04-13

**Authors:** Nicolas S. Baumann, Nicole Torti, Suzanne P. M. Welten, Isabel Barnstorf, Mariana Borsa, Katharina Pallmer, Jennifer D. Oduro, Luka Cicin-Sain, Koichi Ikuta, Burkhard Ludewig, Annette Oxenius

**Affiliations:** 1 Institute of Microbiology, Department of Biology, ETH Zürich, Zürich, Switzerland; 2 Department of Vaccinology and applied Microbiology, Helmholtz Centre for Infection Research, Braunschweig, Germany; 3 Laboratory of Immune Regulation, Department of Virus Research, Institute for Frontier Life and Medical Sciences, Kyoto University, Kyoto, Japan; 4 Institute of Immunobiology, Kantonsspital St. Gallen, St. Gallen, Switzerland; Oregon Health Sciences University, UNITED STATES

## Abstract

Cytomegalovirus (CMV) infection induces an atypical CD8 T cell response, termed inflationary, that is characterised by accumulation and maintenance of high numbers of effector memory like cells in circulation and peripheral tissues—a feature being successfully harnessed for vaccine purposes. Although stability of this population depends on recurrent antigen encounter, the requirements for prolonged survival in peripheral tissues remain unknown. Here, we reveal that murine CMV-specific inflationary CD8 T cells are maintained in an antigen-independent manner and have a half-life of 12 weeks in the lung tissue. This half-life is drastically longer than the one of phenotypically comparable inflationary effector cells. IL-15 alone, and none of other common γ-cytokines, was crucial for survival of inflationary cells in peripheral organs. IL-15, mainly produced by non-hematopoietic cells in lung tissue and being trans-presented, promoted inflationary T cell survival by increasing expression of Bcl-2. These results indicate that inflationary CD8 T cells are not just simply effector-like cells, rather they share properties of both effector and memory CD8 T cells and they appear to be long-lived cells compared to the effector cells from acute virus infections.

## Author summary

A majority of the human population is infected with cytomegalovirus (CMV), which results in lifelong persistence due to viral latency. CMV induces remarkably strong and sustained effector memory-like CD8 T cell responses in circulation and peripheral tissues, also referred to as memory CD8 T cell "inflation". In tissues, these effector memory-like cells contribute to immunosurveillance and early control of CMV reactivation events. Due to the high numbers and effector-like functional properties of inflationary CD8 T cells in peripheral tissues, CMV-based vectors are gaining substantial interest in the context of T cell based vaccines that protect peripheral tissues against infections or tumors. Here, we investigated how the stable peripheral pool of inflationary CD8 T cells is maintained and show that inflationary CD8 T cells are long-lived T cells and have a markedly prolonged half-life compared to effector CD8 T cells. In peripheral organs such as lung, IL-15 cytokine is pivotal in promoting maintenance of inflationary cells by inducing expression of the anti-apoptotic molecule Bcl-2. We show that IL-15 is mainly expressed by non-hematopoietic cells in lung tissue and that IL-15 is trans-presented to the inflationary CD8 T cells in vivo. Thus, CMV-driven inflationary CD8 T cell responses represent a unique T cell subset in peripheral tissues that is regulated differently compared to T_RM_ CD8 T cells emerging after vaccination or acute infections.

## Introduction

T cells are a major part of the adaptive immune system and have the ability to differentiate into memory T cells after previous stimulation with cognate antigen. Memory T cells can react much faster upon antigen restimulation by re-expansion and immediate effector functions. The induction of memory T cells, in particular of memory T cells that reside long-term in peripheral tissues, is of considerable interest for the development of T cell-based vaccines, as they afford protection *in situ* in case of local pathogen infection. Acute infections induce memory T cells, which mainly reside in lymphoid tissues, even long after pathogen clearance [1, 2]. These are defined as central-memory T cells (T_CM_) expressing CD62L, CD127 (IL-7Rα) and CCR7 [1, 2]. Aside T_CM_, acute infections also induce effector-memory T cells (T_EM_, CD62L^-^, IL-7Rα^+^ and CCR7^-^), mainly at early time points after resolution of the infection, and the more recently discovered tissue-resident memory T cells (T_RM_) that can permanently lodge into tissues, are disconnected from the circulation [3] and exhibit protection during local reinfection events [4–6]. Despite the fact that acute viral infections and / or vaccinations are able to induce T_RM_ cells, there are specific vectors that excel in the ability to induce large populations of effector-like memory cells in peripheral tissues. Cytomegalovirus, a member of the herpes virus family, is a potent inducer of a very large memory T cell pool, consisting of distinct memory subsets with different localisations, phenotypes and functions. In various species, CMV infections induce an exquisitely large and sustained population of functional memory CD8 T cells residing in peripheral tissues [7–11]. Thus, CMV-based vectors have gained broad interest in the development of T cell-based vaccines. Indeed, recombinant CMV-based vaccine vectors expressing SIV- or ebolavirus glycoprotein-derived epitopes showed protection of vaccinated rhesus macaques against SIV and ebolavirus challenge. Also, vaccinations with recombinant murine CMVs (MCMVs) expressing influenza A NP epitope provided protection of mice against VacV-NP challenge [12–16].

The sustained expansion and maintenance of CMV-specific CD8 T cells in peripheral tissues is a hallmark of CMV infection and accounts only for CMV-reactive CD8 T cells with certain specificities. While some CD8 T cells with defined specificities are differentiating along the "classical" expansion / contraction / memory formation pathway, specific CD8 T cell subsets follow a substantially different kinetics and differentiation trajectory. These CMV-specific CD8 T cells continue to expand also at time points when their "classical" counterparts decline in numbers, and establish themselves as a stable population of memory CD8 T cells with an activated phenotype in peripheral tissues [8, 17–19]. Importantly, these activated memory CD8 T cells residing in peripheral tissues exhibit an exquisite capacity to control peripheral infections [20, 21]. The mechanisms responsible for this "inflationary" behaviour of specific CMV-reactive CD8 T cell populations are currently still ill-defined. However, a number of studies have elaborated that CMV antigen presentation, resulting from CMV reactivation events, drives memory CD8 T cell inflation and that the ability of a given epitope to induce memory inflation depends on the context of gene expression and its processing by the conventional immunoproteasome [22–26]. Inflationary CD8 T cells exhibit high expression levels of the terminal differentiation marker KLRG-1 and are highly functional with respect to pro-inflammatory cytokine production and cytotoxicity, unlike functionally exhausted cells emerging in the context of highly active chronic viral infections [18, 21, 27–29]. Inflationary CD8 T cells were described as short-lived effector cells whose pool needs to be continuously replenished to maintain stable numbers in peripheral tissues [11]. This replenishment relies on reactivation and re-expansion of T_CM_ cells by cognate antigen presented on non-hematopoietic cells—presumably derived from viral reactivation events—taking place in lymph nodes or in the vasculature [25, 30, 31]. However, it remains unclear how long these reactivated cells persist in peripheral tissues and how peripheral maintenance of inflationary T cells is regulated.

In this study, we assessed these questions using a monoclonal MCMV-specific CD8 T cell population specific for an inflationary epitope in peripheral organs. Our data demonstrate that inflationary CD8 T cells in the lung exhibit a half-life between 10–12 weeks, independent of local antigen. We reveal a decisive role of IL-15 in maintaining inflationary T cells in the lungs, whereas all other common γ-chain cytokines are dispensable. We identified a dominant role of IL-15 production in non-hematopoietic cells in the lung for the maintenance of lung-resident inflationary CD8 T cells, which also bears relevance for the use of CMV-based vectors for vaccine purposes.

## Results

### Long-term maintenance of inflationary MCMV-specific CD8 T cells in peripheral organs during latent MCMV infection

**Fig 1:**
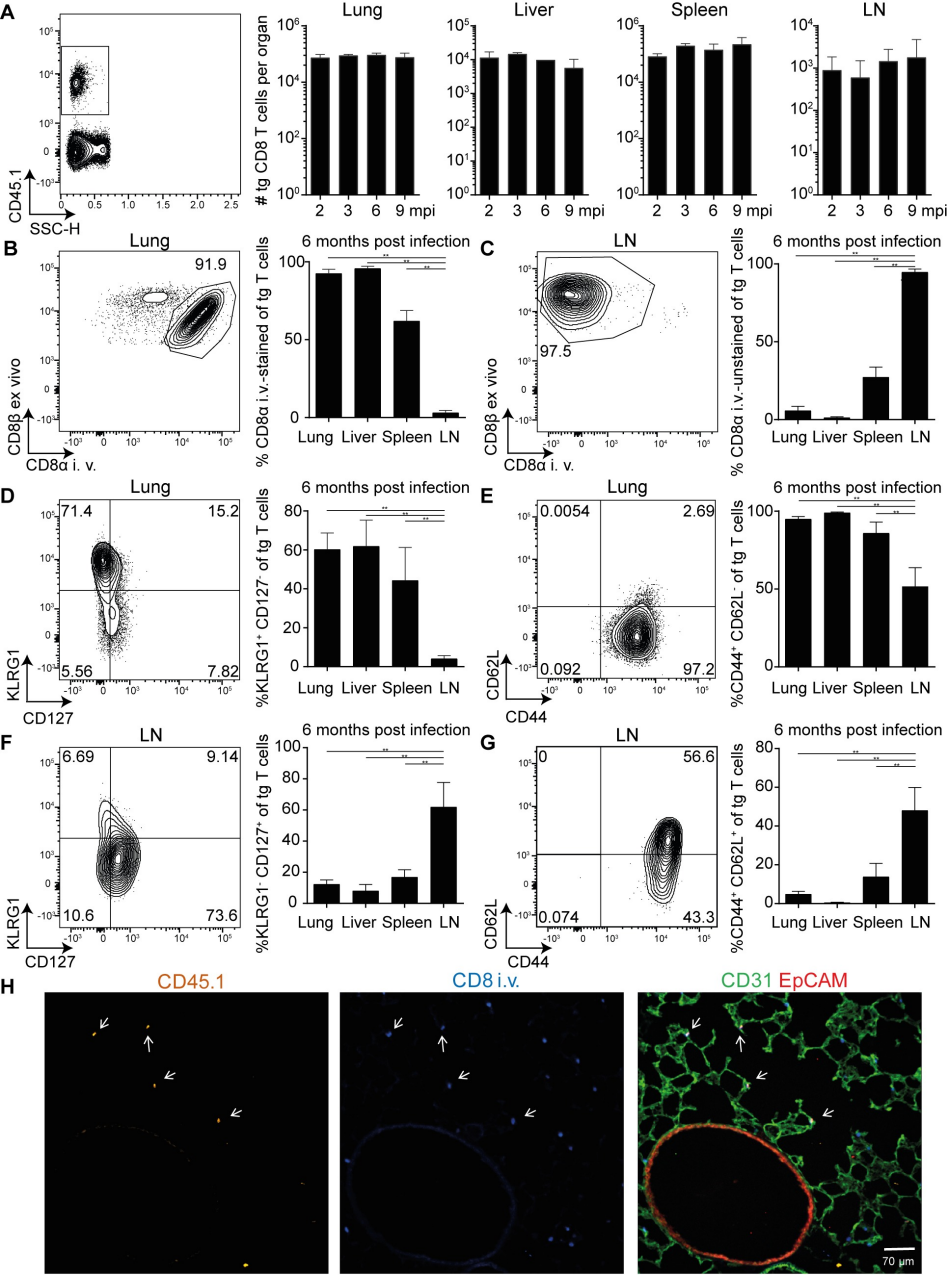
Transgenic Maxi CD8 T cells are maintained at high numbers during latency and exhibit an effector-memory phenotype 10^4^ naïve Maxi CD8 T cells were adoptively transferred into naïve C57BL/6J mice followed by i. v. MCMV infection. Mice were injected i. v. with anti-CD8α antibody three minutes prior to euthanasia. (A) Representative flow cytometry contour plot gating on Maxi cells and their total numbers are depicted in the lungs, liver, spleen and LN at 2, 3, 6 and 9 months post infection and are shown as mean + SEM of n = 3 mice, representative of two independent experiments. Representative flow cytometry contour plots of Maxi cells in the lung (B) and LN (C), and percentage of CD8α-stained Maxis (B), and CD8α-unstained Maxi cells (C) at 6 months post infection are shown. Representative contour plot of KLRG1 and CD127 (D, F), and CD44 and CD62L (E, G) expression on Maxi cells in the lung (D,E) and LNs (F,G) and mean percentages are shown. Representative contour plot of Maxi cell expression of KLRG-1 and CD127 (D, F), and CD44 and CD62L (E, G) in the lung, mean percentages are shown. (F)Data (B-G) are shown as mean + SEM of n = 6 mice pooled from two independent experiments. (A-G) **p<0.01. Statistical analyses were performed using the non-parametric Mann-Whitney *U* test. (H) Maxi cells are localized in the proximity of CD31^+^ endothelial cells in the lungs. Naïve CD45.1 Maxi CD8 T cells were adoptively transferred into WT mice one day prior to i.v. infection with MCMVΔm157. Mice were injected i. v. with 5 μg anti-CD8α-BV421 antibody 3 minutes prior to euthanasia. The localization of Maxi cells in the lungs was determined in the latent phase of MCMV infection. CD45.1 is shown in orange, blue identifies CD8 T cells that were stained upon i.v. injection of αCD8 antibody, CD31 is shown in green and EpCAM in red. Arrows indicate CD45.1^+^ Maxi cells that are additionally stained by the i.v. injected CD8 antibody.

To assess the long-term maintenance of inflationary MCMV-specific CD8 T cells in peripheral organs, we adoptively transferred M38-specific TCR transgenic CD8 T cells (Maxi) into naïve C57BL/6 hosts, followed by MCMV infection. We analysed frequencies and total numbers and phenotype of transgenic Maxi cells at various time points post infection in several organs after MCMV had established latency. We found that the total number of Maxi CD8 T cells was kept constant in all organs up to nine months post infection (Fig 1A). Performing intravascular labelling of Maxi cells revealed that high frequencies of Maxi cells resides either within or in close contact to the circulation in lungs, liver and spleen, but not in lymph nodes (Fig 1B and 1C).

In accordance with previous studies, a vast majority of inflationary cells in lung, liver, spleen and lymph nodes does not express the typical T_RM_ markers CD69 or CD103 (S1 Fig), but expresses markers associated with effector-like T cells (CD127^low^, KLRG-1^+^, CD62L^-^, CD44^+^), particularly in the lung, liver and spleen at all time points analysed (Fig 1D and 1E) [32, 33]. In contrast, the majority of Maxi cells present in the lymph nodes express markers associated with central-memory T cells (CD127^+^, KLRG-1^-^, CD62L^+^, CD44^+^), confirming previous data on endogenous M38-specific CD8 T cells (Fig 1F and 1G). Therefore, we conclude that transgenic Maxi cells display similar properties with respect to number and phenotype as described for endogenous inflationary CD8 T cells.

Furthermore, to determine more precisely the location of M38-specific Maxi cells in lung tissue, we performed fluorescence microscopy on lung sections. Maxi cells, identified by CD45.1 staining, were located close to CD31^+^ endothelial cells and were not found in association with EpCAM^+^ epithelial cells (Fig 1H). Furthermore, the majority of the CD45.1 Maxi cells were labelled by i.v. administered αCD8 antibody, marked by white arrows.

### Maintenance of inflationary Maxi cells in the periphery is antigen-independent

**Fig 2:**
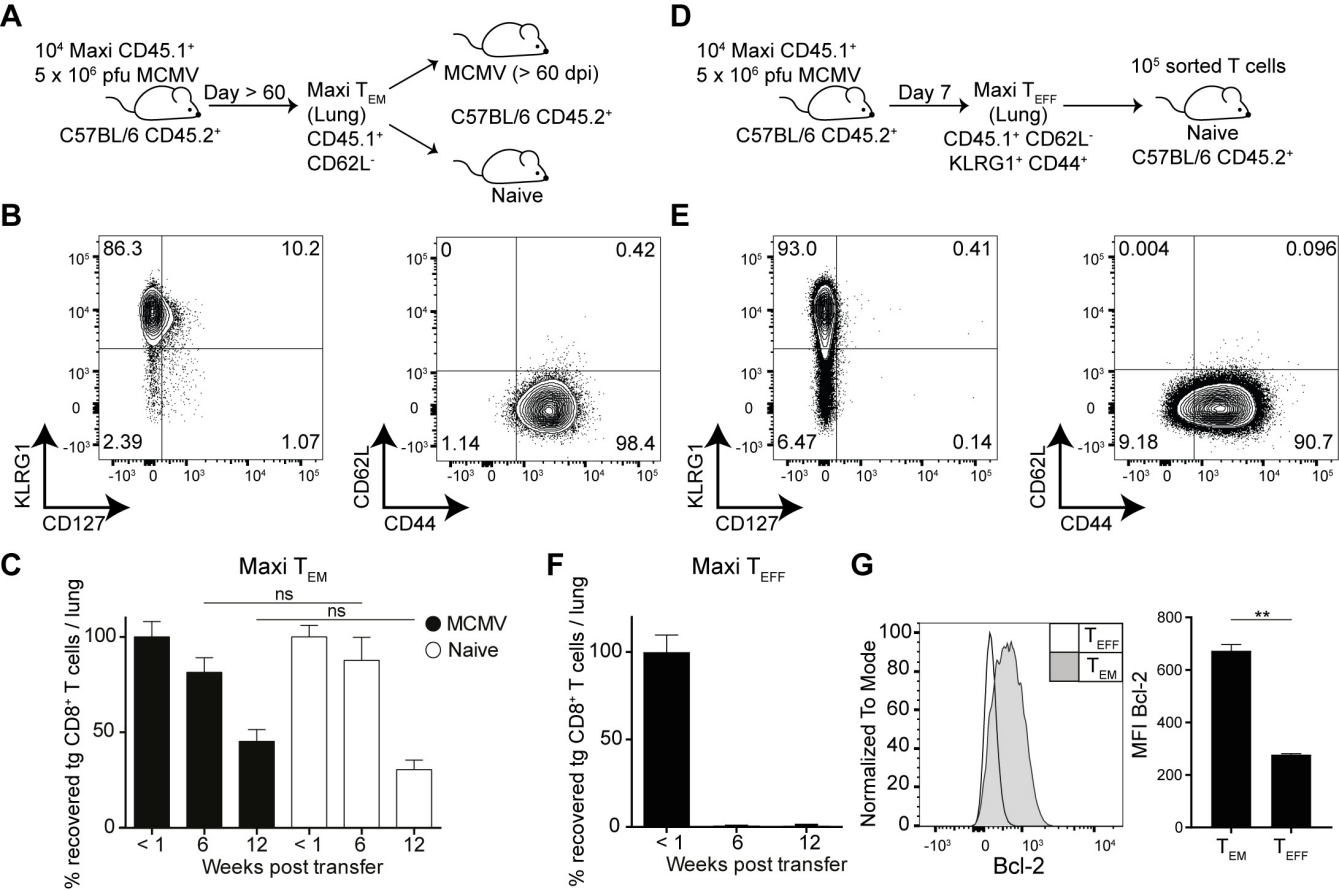
Effector-memory Maxi CD8 T cells are maintained in lungs independently of antigen and have longer half-lives than effector Maxi CD8 T cells. (A) Naïve Maxi CD8 T cells were adoptively transferred into naïve C57BL/6 mice followed by i. v. MCMVΔm157 infection. Effector-memory Maxi CD8 T cells were sorted from the lungs and transferred into infection-matched or naïve recipients. Total numbers of Maxi cells were assessed in the lungs at <1, 6 and 12 weeks post transfer. (B) Representative contour plots showing Maxi T cell phenotype from the lung tissue (> 60 dpi). (C) Percentage transgenic Maxi cells recovered from the lung are shown normalized to the total numbers recovered within the first week post transfer. Data are shown as mean + SEM of n = 13–17 mice pooled from five independent experiments. (D) Experimental setup: Naïve Maxi CD8 T cells were adoptively transferred into naïve C57BL/6 mice followed by i. v. MCMVΔm157 infection. Effector Maxi CD8 T cells were sorted from the lungs (7 dpi) and transferred into naïve recipients. (E) Representative contour plots showing Maxi T cell phenotype from the lung tissue (7dpi). (F) Percentage transgenic Maxi cells recovered from the lung is shown normalized to the total numbers recovered within the first week post transfer. Data are shown as mean + SEM of n = 10 mice pooled from two independent experiments. (G) Representative Bcl-2 histogram from effector and effector-memory Maxi CD8 T cells. Mean fluorescence intensities are shown + SEM of n = 6 mice pooled from two independent experiments. (C; E-F) ns, not significant; **p<0.01. Statistical analyses were performed using the non-parametric Mann-Whitney *U* test.

Since sporadic reactivation events and antigen presentation on non-hematopoietic cells are essential for driving memory inflation during MCMV latency, it is likely that the peripheral pool of inflationary CD8 T cells is continuously fuelled by recently re-activated T_CM_ Maxi cells. Hence, the inflationary pool would decline when the T_CM_ pool would be ablated. We tested this hypothesis in an adoptive transfer system where we transplanted T_EM_ Maxi T cells isolated from lungs of latently infected mice into infection-matched or naïve C57BL/6J recipient mice. Specifically, at 60 days post infection, we isolated lymphocytes from the lung tissues and sorted effector-memory Maxi T cells based on CD44^+^ and CD62L^-^ expression (Fig 2A and 2B). In the recipients, total numbers of Maxi cells recovered from the lung tissue were quantified at three different time points to determine the half-life of the inflationary T cell pool. We found that transferred Maxi cells exhibited comparable half-lives of 10–12 weeks in lungs of naïve or infection-matched recipients, indicating that survival of inflationary cells in lungs is independent of antigen (Fig 2C). This half-life is slightly longer compared to previous publications where phenotypically mixed inflationary cells from the spleen were described to have a half-life of 45–60 days [11]. Of note, comparable half-lives to lung tissue were observed in spleen (S2 Fig).

Being surprised by the relatively long half-life of effector-memory Maxi cells, we asked the question how phenotypically similar effector cells from an acute time point of infection would compare with respect to their half-lives to inflationary cells from the latent phase of infection. We therefore sorted CD44^+^ CD62L^-^, KLRG1^+^ Maxi CD8 T cells from lungs of acutely MCMV infected mice (day 7, Fig 2D and 2E), transferred them into naïve recipients and quantified Maxi cells <1 week, 6 and 12 weeks post transfer in the lungs of the recipients. These Maxi cells isolated from day 7 post infection had a strikingly shorter half-life, less than 6 weeks, despite expressing similar surface markers as the cells sorted from latent MCMV infection (Fig 2F). As inflationary M38-specific CD8 T cells steadily increase their levels of the anti-apoptotic protein Bcl-2 over the course of the MCMV infection [25], we compared Bcl-2 levels in day 7 and day 60 Maxi cells. Indeed the levels of Bcl-2 were significantly higher in effector-memory Maxi cells from latently infected mice compared to effector cells from acute infection (Fig 2G), suggesting that inflationary T_EM_ and inflationary T_EFF_ CD8 T cells differ with respect to their longevity.

**Fig 3:**
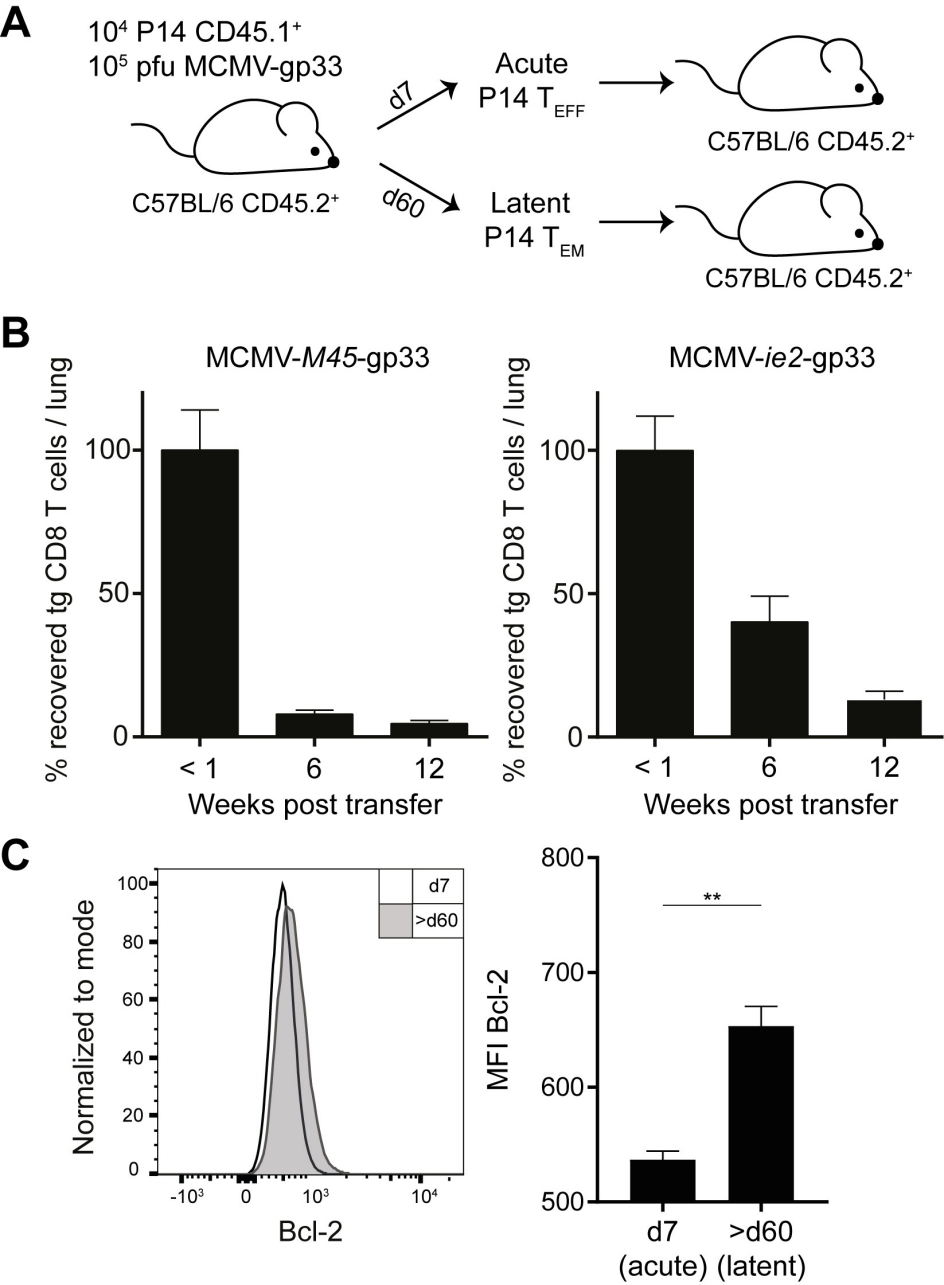
Inflationary T_EM_ P14 cells have a longer half-life than effector P14 CD8 T cells. (A) Naïve P14 CD8 T cells were adoptively transferred into naïve C57BL/6 mice followed by i. v. infection with MCMV-M45-gp33 for acute P14 T_EFF_ or MCMV-ie2-gp33 for latent P14 T_EM_. Effector P14 T cells (7 dpi) and T_EM_ P14 cells (> 60 dpi) were sorted from the lungs and transferred into naïve recipients. (B) Left graph: Effector P14; Right graph: T_EM_ P14. Percentage recovered P14 cells from the lungs normalized to <1 week time point is shown as mean + SEM of n = 10 mice pooled from two independent experiments. (C) Representative histograms of T_EFF_ and T_EM_ P14 T cell population from the lung tissues are shown. Mean fluorescence intensities of Bcl-2 of P14 cells (7 dpi) and P14 (> 60 dpi) are shown as mean + SEM of n = 4–5 mice representative from two independent experiments. (A, B); **p<0.01. Statistical analyses were performed using the non-parametric Mann-Whitney U test.

As effector Maxi cells had a different half-life compared to inflationary effector-memory Maxi cells, we wondered whether this observation also holds for CD8 T cells with a different specificity. We made use of the TCR transgenic P14 mouse, whose CD8 T cells are all specific for the gp_33-41_ epitope of lymphocytic choriomeningitis virus (LCMV) in combination with recombinant MCMVs that express gp33 under the *ie2* promotor (inflationary kinetics) or the *M45* promotor (non-inflationary kinetics) [34, 35]. Naïve P14 cells were transferred into naïve recipients prior to MCMV-gp33 infection. P14 effector memory cells were sorted from lungs on day 7 post MCMV-*M45*-gp33 infection, and at least 60 days post MCMV-*ie2*-gp33 infection, and further transferred them into naïve recipients (Fig 3A). Notably, we corroborated the results obtained with Maxi cells, such that effector P14 cells had a diminished half-life when compared to the T_EM_ P14 counterparts (Fig 3B). The shorter half-life was once more correlated with reduced Bcl-2 levels in comparison to T_EM_ P14 cells, suggesting an important role of Bcl-2 in the maintenance and survival of inflationary T cells during latent MCMV infection.

### Inflationary T cells require IL-15 survival signals for their maintenance

T cells and particularly memory CD8 T cell were shown to be maintained by homeostatic cytokines. Moreover, the continuous upregulation of the anti-apoptotic protein Bcl-2 over the course of MCMV infection indicates that potential exposure to homeostatic cytokines in the organs might promote T cell survival [25, 36, 37, 38].

**Fig 4:**
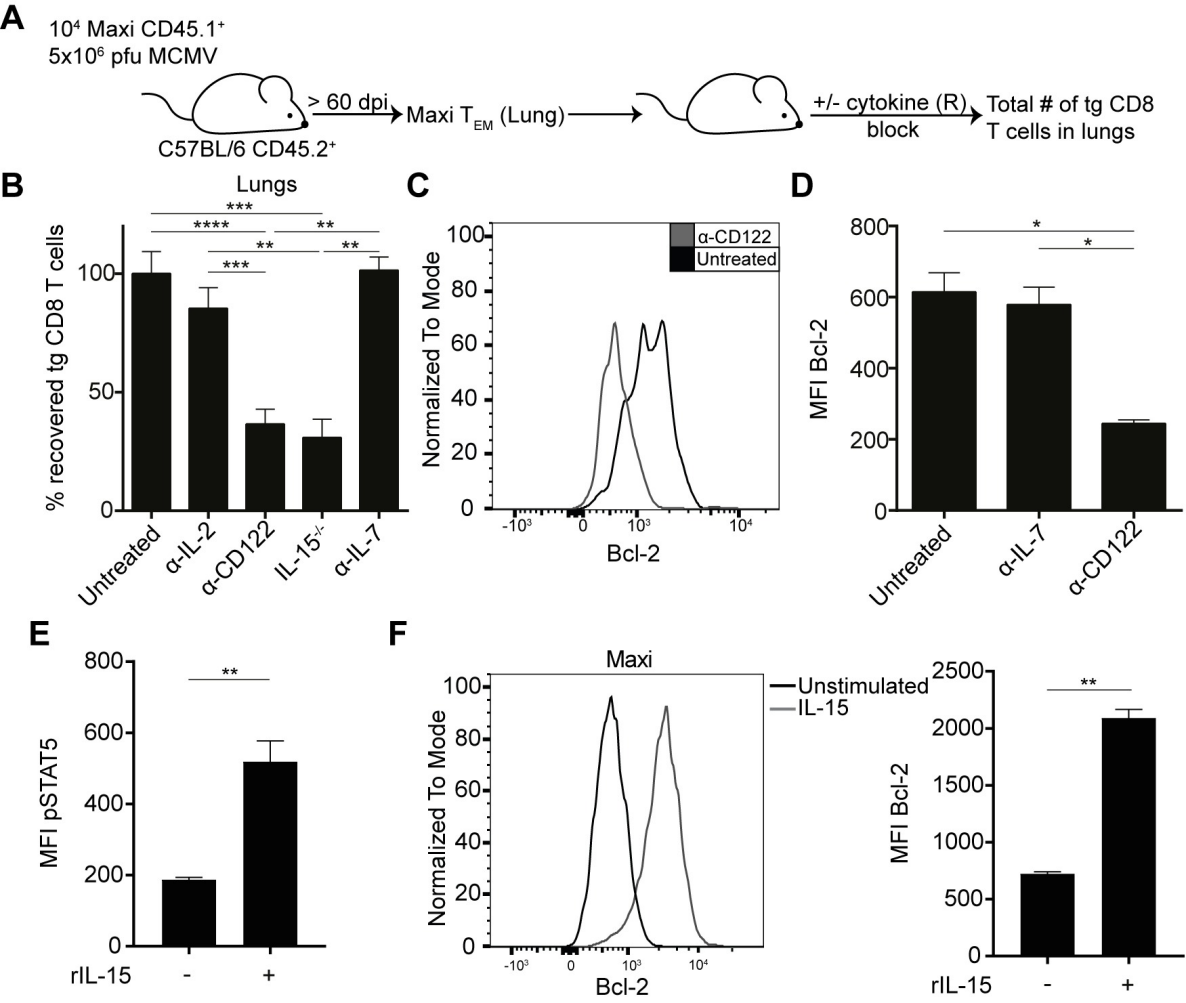
Maintenance of effector-memory Maxi CD8 T cells is dependent on IL-15 but independent of IL-7 or IL-2. (A) Naïve Maxi CD8 T cells were adoptively transferred into naïve C57BL/6 mice followed by i. v. MCMVΔm157 infection. Effector-memory Maxi T cells were sorted from the lungs and transferred into C57BL/6 recipients or knockout recipients. Recipients were administered i.p. during 30 days with receptor-blocking or cytokine neutralising antibodies every second day. Total numbers of Maxi cells were assessed in the lungs at 4 weeks post transfer. (B) Percentage of recovered Maxi cells normalized to untreated control is shown as mean + SEM of n = 4–12 mice pooled from five independent experiments. (C) Representative Bcl-2 histogram gated on Maxi CD8 T cells. (D) Mean fluorescence intensities of Maxi CD8 T cells are shown as mean + SEM of n = 4 mice representative of two independent experiments. (E) Mean fluorescence intensities of pSTAT5 in inflationary Maxi cells are shown as mean + SEM of n = 6 mice pooled from two independent experiments. (F) Representative Bcl-2 histogram gated on Maxi CD8 T cells and mean fluorescence intensities of Bcl-2 in inflationary Maxi cells are shown as mean + SEM of n = 6 mice pooled from two independent experiments. (B, D-F) *p<0.05; **p<0.01; ***p<0.001; ****p<0.0001. Statistical analyses were performed using the non-parametric Mann-Whitney *U* test.

To address the question whether and which homeostatic cytokines might promote the survival of peripheral inflationary CD8 T cells, inflationary Maxi cells were generated as described before, sorted from lung tissues, and adoptively transferred into infection matched C57BL/6 mice. The recipients were continuously treated with cytokine / cytokine receptor blocking antibodies for a period of 30 days before quantification of Maxi cells in lung tissue. Alternatively, inflationary Maxi T_EM_ cells from lungs were transferred into naïve cytokine-deficient recipients and remaining Maxi cells were also quantified 30 days post transfer (Fig 4A). Interestingly, we observed no difference in recipients that were treated with neutralising α-IL-7 antibodies (Fig 4B). Also, we found no evidence for a role of IL-2 as mice treated with neutralising α-IL-2 antibodies showed no significant reduction in Maxi numbers in the lung. Strikingly, the only cytokine that had an influence on the maintenance of inflationary T_EM_ Maxi cells was IL-15. Namely, when recipient mice were either administered with receptor blocking α-CD122 (IL-2/15Rβ) antibody or Maxi cells were adoptively transferred into IL-15-deficient mice, we observed a significantly reduced recovery of Maxi cells from the lung tissue (Fig 4B). We hypothesized that the reduced numbers of Maxi cells were associated with reduced Bcl-2 expression in IL-15-deficient recipients. Indeed, significantly reduced levels of Bcl-2 were apparent when the mice were treated with α-CD122 antibodies or when the recipients were IL-15 deficient (Fig 4C and 4D). No reduced levels of Bcl-2 were observed in any other conditions (Fig 4D). We exposed inflationary Maxi cells *in vitro* to recombinant IL-15 and found upregulation of pSTAT5 (Fig 4E) and Bcl-2 (Fig 4F) highlighting that inflationary cells are responsive to IL-15. These findings suggest a pivotal role for IL-15 in the maintenance of inflationary CD8 T cells, most likely by enhancing survival via upregulation of the anti-apoptotic protein Bcl-2.

As IL-15 neutralization or use of IL-15 deficient recipient mice might also target other immune cells than the transferred T_EM_ Maxi cells—in particular NK cells that are highly dependent on IL-15 signalling, we wanted to rule out a potential involvement of NK cells in the maintenance of inflationary CD8 T cells. Therefore, we adapted our transfer model including administration of α-NK1.1 antibodies over four weeks and assessed the total numbers of surviving Maxi cells in the lung tissues (S3A Fig). We did not detect significant differences in total numbers of Maxi cells in any of the organs analysed (S3B Fig), suggesting that IL-15 acts directly on inflationary T cells. To control for NK cell depletion, we enumerated the total numbers of NK cells in the lungs and observed a strong reduction in the NK cell numbers in all organs analysed (S3C Fig). We therefore concluded that IL-15 has a direct role in providing survival signals to inflationary CD8 T cells.

### IL-15 deficiency leads to reduced memory inflation during MCMV infection

**Fig 5:**
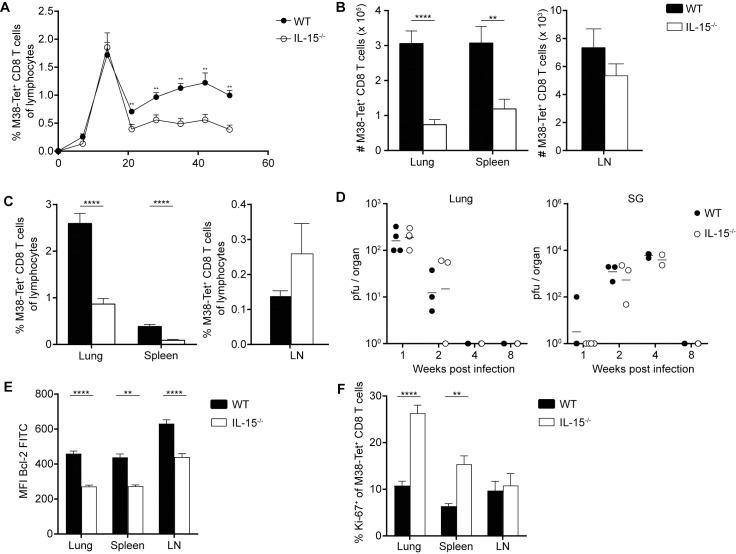
IL-15-deficient mice have reduced inflationary CD8 T cell maintenance. (A) Naïve C57BL/6 mice and IL-15^-/-^ mice were infected i. v. with MCMVΔm157. Percentages of M38-specific CD8 T cells of total lymphocytes were longitudinally measured in the blood. (B) Total number of M38-specific CD8 T cells and (C) percentages of M38-specific CD8 T cells of total lymphocytes in lungs, spleen and LNs at 62 dpi are shown as mean + SEM of n = 8–10 mice representative from three independent experiments. (D) Viral titres of infected C57BL/6 and IL-15^-/-^ mice are shown as mean of n = 2–4 mice representative of two independent experiments. (E) Mean fluorescence intensities of Bcl-2 on M38-specific CD8 T cells are shown as mean + SEM of n = 8–10 mice representative from three independent experiments. (F) Percentage of Ki67^+^ of M38-specific CD8 T cells in lungs, spleen and LNs at 62 dpi are shown as mean + SEM of n = 8–10 mice representative from three independent experiments. (A-E); **p<0.01; ****p<0.0001. Statistical analyses were performed using the non-parametric Mann-Whitney *U* test.

To confirm a pivotal role of IL-15 in sustaining memory CD8 T cell inflation during MCMV infection, we infected naïve C57BL/6 and IL-15^-/-^ mice with MCMV and followed endogenous M38-specific CD8 T cells over the course of infection in the blood. Interestingly, we did not observe differences in percentages of M38-specific CD8 T cells during acute MCMV infection, suggesting that priming of naïve CD8 T cells and clonal expansion are not affected in the absence of IL-15. Yet, when MCMV latency was established and lytic virus replication was absent from most peripheral organs except the salivary glands, IL-15-deficient animals had significantly reduced frequencies of M38-specific CD8 T cells compared to wild type mice (Fig 5A) despite comparable control of lytic MCMV infection (Fig 5D). Detailed analysis of the abundance of virus-specific cells during latency in the organs revealed organ-specific reductions in total numbers and percentages of M38-specific CD8 T cells in the lungs and spleen, whereas in the LNs there were no significant differences observed (Fig 5B and 5C). With most T_CM_ cells specific for M38 being located in lymphoid tissues, particularly in LNs, this observation suggests that maintenance of T_CM_ M38-specific CD8 T cells does not critically depend on IL-15 signals in contrast to their peripheral T_EM_ counterparts. Consistent with previous experiments, Bcl-2 levels in M38-specific CD8 T cells were reduced in IL-15-deficient hosts in all organs (Fig 5E). Interestingly, we found a higher percentage of Ki-67^+^ M38-specific CD8 T cells in the lungs and spleen of IL-15 deficient hosts, but not in the LNs (Fig 5F). It is conceivable that increased peripheral antigen encounter due to inferior MCMV reactivation surveillance by reduced numbers of T_EM_ cells might be responsible for this increased exit from G0 stage in M38-specific CD8 T cells.

### IL-15 ablation in non-hematopoietic cells reduces inflationary T cell maintenance

So far, we could unravel a crucial role for IL-15 in maintaining the peripheral inflationary T cell pool in lung tissue. However, it remained an open question, which cellular source is responsible for providing IL-15. Several cellular sources have been described to be able to express IL-15, including macrophages, dendritic cells as well as stromal cells [39–41].

**Fig 6:**
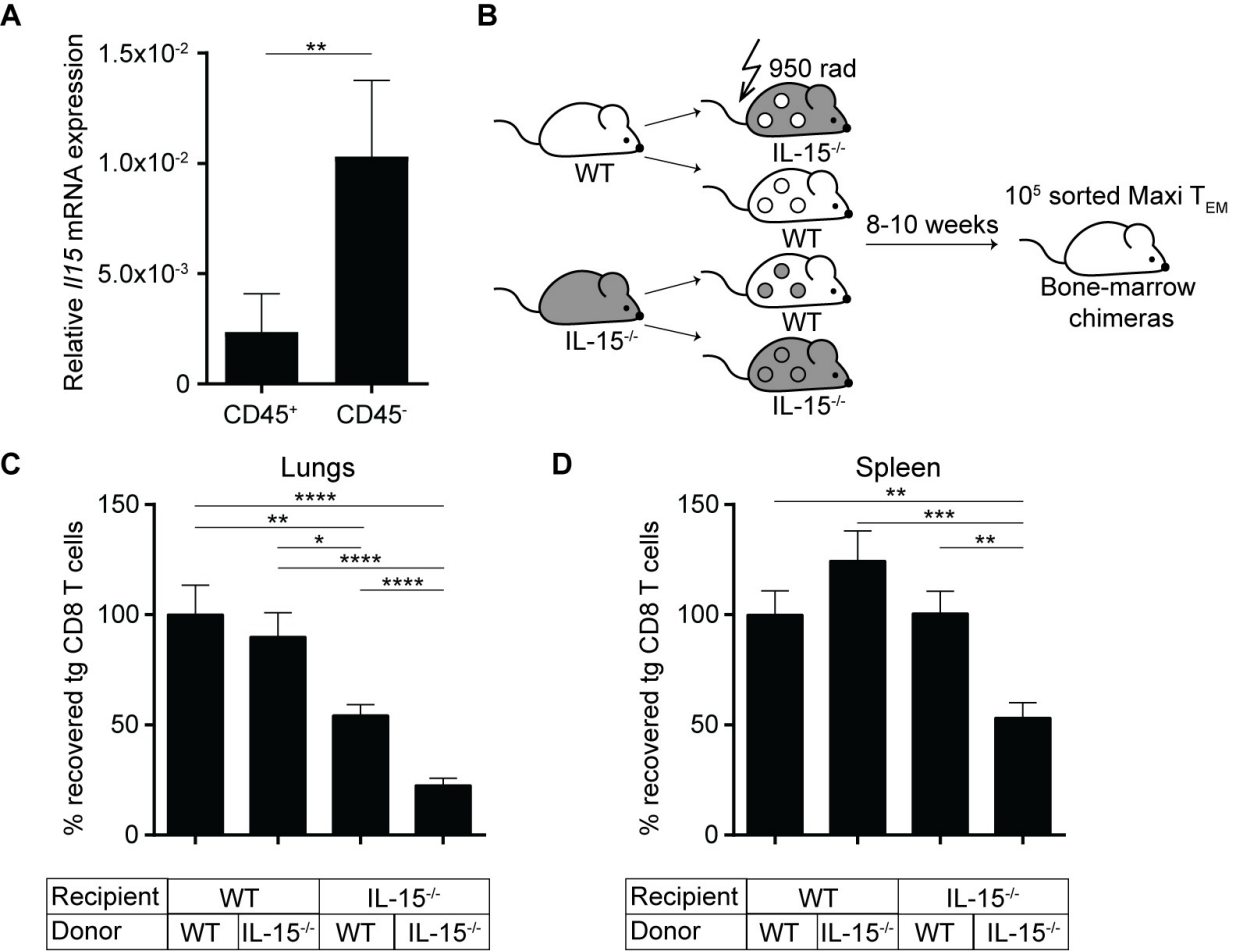
IL-15 ablation in non-hematopoietic cells reduces inflationary T cell maintenance. (A) Lung lymphocytes were isolated from naïve C57BL/6 mice and sorted according to CD45 staining. Relative *Il15* mRNA levels normalized to β-actin internal controls was calculated using the ΔΔC_T_ method, shown as mean + SEM of n = 6 mice pooled from two independent experiments. (B) Experimental setup: Naïve IL-15^-/-^ and Thy1.1 mice were lethally irradiated and reconstituted with indicated bone marrow cells. Inflationary Maxi T_EM_ cells were sorted from lungs of latently infected donor mice and transferred into the chimeras. The total numbers of Maxi cells were assessed four weeks post transfer. Total numbers of Maxi CD8 T cells in lungs (C), and spleen (D) at four weeks post transfer are shown as mean + SEM of n = 12–15 mice pooled from three independent experiments. (A; C-D); *p<0.05; **p<0.01; ***p<0.001; ****p<0.0001. Statistical analyses were performed using the non-parametric Mann-Whitney *U* test.

Hence, we quantified IL-15 expression levels in various cellular compartments of lung tissue. We isolated lung tissue of naïve C57BL/6 mice and sorted cells into hematopoietic and the non-hematopoietic origin by staining for CD45, isolated RNA and determined the levels of *Il15* mRNA by RT-qPCR. We found that the relative *Il15* expression level was higher in the non-hematopoietic compartment compared to the hematopoietic compartment (Fig 6A), indicating a potential role of IL-15 production by non-hematopoietic cells in the maintenance of inflationary T cells. To confirm a potential involvement of non-hematopoietic cells *in vivo*, we generated bone-marrow chimeric mice with selective IL-15 deficiency in either hematopoietic or non-hematopoietic compartments (Fig 6B). Chimerism of reconstituted mice was confirmed (S4 Fig).

We then sorted inflationary Maxi T_EM_ cells from latently infected donor mice and adoptively transferred them into the chimeras. Four weeks post transfer we assessed the total numbers of recovered Maxi cells in the lung and in the spleens. In the lungs (Fig 6C), we observed the lowest recovery of Maxi cells in mice where both non-hematopoietic and hematopoietic cells were deficient of IL-15, consistent with previous experiments performed in IL-15^-/-^ mice. Interestingly, we observed a 50% reduced recovery in mice lacking IL-15 on non-hematopoietic cells, whereas in IL-15-deficient hematopoietic cells no difference was observed (Fig 6C). In the spleen, none of the chimeras which selectively lacked IL-15 in one or the other compartment showed a reduced recovery of Maxi cells, only the complete deficiency resulted in a reduced Maxi cell number (Fig 6D). These results suggested an organ-specific role of non-hematopoietic cells in expression of IL-15, promoting maintenance of inflationary T cells.

### Cell-type specific IL-15 deficiency had little influence on inflationary T cell maintenance

Next, we aimed to identify the specific cellular source of IL-15 for the maintenance of inflationary M38-specific CD8 T cells in lung tissue. Thus, we analysed *Il15* mRNA levels in different stromal cells from the lungs of naïve C57BL/6 mice. However, we were unable to identify a cell type exhibiting dominant IL-15 mRNA production, including epithelial cells, blood endothelial cells nor lymphoid endothelial cells (S5 Fig).

Next, we quantified the maintenance of inflationary T cells in mice that lacked IL-15 expression in specific cell types. We used mice with cell-type specific depletion of IL-15, including CCL19-producing fibroblastic reticular cells (FRCs) (Ccl19-Cre *Il15*^fl/fl^), CD11c-expressing dendritic cells and alveolar macrophages (CD11c-Cre *Il15*^*fl*/fl^), and endothelial cells (*VE-Cadherin-*Cre *Il15*^fl/fl^). The reasons for these choices are that 1) FRCs were shown to be involved in control ILC1 cells through IL-15 in the intestine [41]. 2) Dendritic cells and macrophages have been shown to be important to support effector-memory and central-memory T cells through IL-15Rα, trans-presenting IL-15 to the responding T cells [40]. Also, pulmonary dendritic cells were shown to maintain influenza-specific CD8 T cell responses via IL-15 signals [42]. 3) Endothelial cells are the major cell type of the vasculature and blood vessels and the lung tissue is heavily vascularised, and we observed that inflationary T cells are largely stained by intravascular antibody staining, suggesting localisation in or within close proximity to the vasculature (Fig 1B).

**Fig 7:**
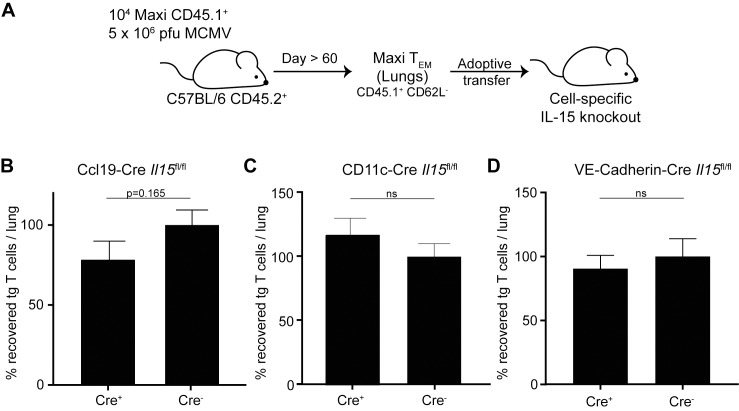
Conditional IL-15 knockout in Ccl19-expressing cells leads to slightly decreased maintenance of inflationary T cells. (A) Naïve C57BL/6 mice were adoptively transferred with naïve Maxi CD8 T cells one day prior to MCMV infection. Maxi T_EM_ cells were sorted from lungs of latently infected donor mice and adoptively transferred into the conditional IL-15 knockout animals. Recovery of Maxi T cells was assessed in the lungs of recipients four weeks post transfer. Percentage of recovered Maxi cells in the lungs normalised to Cre-negative recipients is shown for *Ccl19*-Cre x IL-15^*fl/fl*^ (B) mice as mean + SEM of n = 15–16 mice pooled from three independent experiments. Percentage of recovered Maxi cells in the lungs normalised to Cre-negative recipients is shown in CD11c-Cre *Il15*^*fl*/f*l*^ (C) and VE-Cadherin-Cre *Il15*^*fl*/f*l*^ (D) mice as mean + SEM of n = 7–15 mice pooled from two independent experiments. (B-D) ns, not significant (p>0.20); Statistical analyses were performed using the unpaired, two-tailed Student's *t* test (B-C) or the non-parametric Mann-Whitney *U* test (D).

T_EM_ Maxi cells from lungs of latently infected mice were adoptively transferred into cell-type specific IL-15 knockout mice. We assessed the recovery of Maxi cells from the lung tissue four weeks post transfer (Fig 7A). Neither CD11c-specific ablation of IL-15 (Fig 7C), nor VE-Cadherin-specific deletion of IL-15 (Fig 7D) had an impact on the survival of adoptively transferred T_EM_ Maxi cells. Interestingly, we did observe a small but not significant reduction of recovered Maxi cells in *Ccl19*-Cre x IL-15^*fl/fl*^ mice compared to the Cre-negative control mice (Fig 7B), suggesting a potential role of CCL19-expressing FRCs enhancing inflationary T cell maintenance. However, our data suggest that the source of IL-15 comprises several subtypes of non-hematopoietic as well as some contribution of the hematopoietic cell compartment.

### IL-15Rα is required for inflationary T cell maintenance

IL-15 is usually 'trans-presented' to responding cells by the IL-15-expressing cells, meaning that the expressed IL-15 is bound by the IL-15Rα of the producing cell on its surface, by which it delivers signaling to the responding cell via the CD122(IL-2/15Rβ)/γ_c_ complex [43, 44], mainly being described for dendritic cells and macrophages. We therefore sought to investigate whether trans-presentation of IL-15 by host cells is critical for the survival of transplanted inflationary MCMV-specific CD8 T cells.

**Fig 8:**
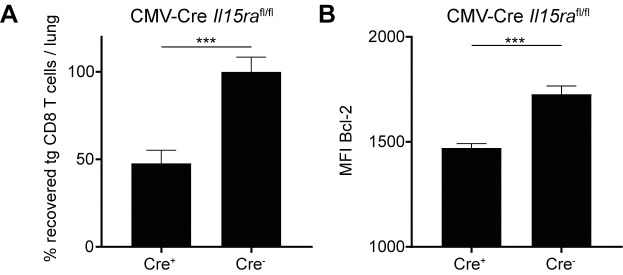
Maintenance of inflationary CD8 T cells is impaired in IL-15Rα deficient hosts. Naïve Maxi CD8 T cells were adoptively transferred into naïve C57BL/6 mice one day prior to MCMV infection. During latency, Maxi T_EM_ cells were sorted from lungs and adoptively transferred into IL-15Rα knockout animals. Recovery of Maxi T cells was assessed in the lungs four weeks post transfer. (A) Percentage of recovered Maxi cells in the lungs normalised to Cre-negative recipients is shown in CMV-Cre *Il15ra*^fl/fl^ mice as mean + SEM of n = 12 mice pooled from two independent experiments. (B) Mean fluorescence intensity of Bcl-2 in Maxi cells from the lungs are shown as mean + SEM of n = 6–8 mice representative of two independent experiments. (A-B) ***p<0.001. Statistical analyses were performed using the non-parametric Mann-Whitney *U* test.

To this end, we crossed *Il15ra*^fl/fl^ mice to a constitutively expressing Cre background (*CMV*-Cre), thereby generating recipient mice that lack IL-15Rα expression. We generated inflationary T cells by transferring naïve Maxi CD8 T cells into naïve C57BL/6 hosts and infected them a day later with MCMV. During viral latency, we isolated lungs, sorted inflationary T_EM_ cells and adoptively transferred them into CMV-Cre *Il15ra*^fl/fl^ mice and Cre-negative control hosts. We assessed the total numbers of Maxi cells recovered from the lung tissue four weeks post transfer. We did observe a significant reduction of recovery of Maxi cells from the lung tissue in CMV-Cre *Il15ra*^fl/fl^ hosts (Fig 8A), suggesting a role of both IL-15 and IL-15Rα by hosts cells to maintain inflationary CMV-specific CD8 T cells. Additionally, we also observed a reduction in the expression of Bcl-2, which is in line with previous findings in IL-15-deficient or CD122-blocked animals (Figs 4C and 5E). Overall, these data suggest an important role of IL-15 during MCMV latency to maintain the inflationary CD8 T cell pool in peripheral organs, in particular by IL-15 / IL-15Rα expressing non-hematopoietic cells.

## Discussion

CMV infection results in a gradual accumulation of inflationary virus-specific CD8 T cells against epitopes that are thought to be expressed in the latent phase during sporadic viral reactivation events [8, 10]. In contrast, non-inflationary CD8 T cells do not inflate, presumably because their cognate antigens are only expressed during acute infection. Also in human CMV (HCMV) infection, studies have demonstrated that particularly in elderly individuals, high frequencies of CMV-specific CD8 T cells accumulate despite absence of lytic virus replication [8, 16, 45]. In this study, we demonstrated that inflationary T cells are kept at comparably high numbers in peripheral organs over nine months post infection exhibiting a remarkably constant phenotype. Whether such high numbers of CMV-specific CD8 T cells are important to assure control of CMV infection is rather unlikely, as CD8-deficient or transiently depleted mice are able to control lytic MCMV infection in peripheral organs, even though there is a moderate increase in virus titres, yet it is likely that there is a higher degree of latent viral loads [21, 46–50]. Also, genetic deletion of an immunodominant inflationary epitope in MCMV resulted in increased and more progressive viral reactivation events in BALB/c mice, although not proceeding to full reactivation and production of infectious virions [51]. Nevertheless, CMV's ability to induce such pronounced numbers of peripherally located CD8 T cells is of great interest for vaccine approaches relying on T cell mediated immunity [12, 14, 52, 53].

Despite a seemingly stable number of inflationary T cells in peripheral organs, there is an underlying dynamic process responsible for this stability. Sensing of CMV-derived antigens on non-hematopoietic cells during viral latency (presumably during early viral reactivation events) and processing of these epitopes by the conventional proteasome were shown to be prerequisites for memory CD8 T cell inflation during MCMV infection [22, 23, 25, 51]. Sensing of CMV-derived antigens by T_CM_ CD8 T cells in secondary lymphoid organs leads to their secondary expansion, effector (memory) differentiation and migration to peripheral tissues, thereby likely contributing to the overall maintenance of the peripheral pool of inflationary CD8 T cells. Consistent with a pivotal role of T_CM_ reactivation in secondary lymphoid organs is the observation that the percentage of Ki-67-expressing M38-specific CD8 T cells is increased in secondary lymphoid organs compared to peripheral organs such as lung or liver during viral latency [11, 25].

Indeed, prior to this study it was shown in adoptive transfer experiments that bulk splenic inflationary T cells have a half-life of 45–60 days [11]. However, in the spleen, there is a considerable variety of phenotypes of inflationary T cells that might influence the measured population half-lives. Here, we quantified the half-life of inflationary M38-specific monoclonal Maxi CD8 T cells in lung tissue—an organ exhibiting pronounced numbers of inflationary CD8 T cells. We demonstrated an antigen-independent maintenance of phenotypically stratified Maxi cells in lung tissue and estimated a half-life of 10–12 weeks that is slightly longer compared to previous data on bulk splenic inflationary T cells [11]. Our data suggest not only that 50% of the inflationary population has to be renewed every 10–12 weeks to guarantee stable numbers, but also that inflationary T cells themselves are rather long-lived, possibly due to relatively high expression levels of the anti-apoptotic molecule Bcl-2. M38-specific effector CD8 T cells from acute MCMV infection exhibit significantly lower levels of Bcl-2 and consistently show a much shorter half-life and poorer maintenance when transferred into naïve recipients compared to their matured inflationary counterparts. Comparable observations were made with effector and inflationary CD8 T cells exhibiting other specificities, where differences in the expression of many other genes such as anti-apoptotic Bcl-_XL_ have been described [54]. Bcl-_XL_ has been associated with the anti-apoptotic effects of IL-15 on the survival of NK cells [55]. Also IL-15 has been demonstrated to induce mRNA expression of Bcl-_XL_ in human PBMCs [56]. These results emphasise that inflationary CD8 T cells, despite sharing many phenotypical markers with effector cells, are not "just" effector cells but a population of activated T cells that have the ability to seed peripheral organs where they are maintained antigen-independently with a relatively long half-life. This notion is also corroborated by transcriptional profiling of effector, memory and inflationary CD8 T cells [54].

Searching for tissue-intrinsic cues that promote relatively long-term survival of inflationary CD8 T cells, we focussed on a potential role of IL-2, IL-7 and IL-15, members of the family of common γ-chain (γ_c_) cytokines that are known to promote differentiation and survival of memory CD8 T cells [57]. Neutralization of these cytokines or their receptors during the maintenance phase of inflationary CD8 T cells revealed a unique role of IL-15 in promoting tissue survival of inflationary CD8 T cells. Sensing of IL-15 by inflationary CD8 T cells *in vivo* was associated with increased Bcl-2 expression, which might directly translate into their increased survival [38, 58–60]. Also, *in vitro*, inflationary T cells exhibited STAT5 phosphorylation and increased Bcl-2 expression upon IL-15 stimulation, highlighting their intact responsiveness to IL-15. Surprisingly, despite IL-7Rα expression on inflationary CD8 T cells, we did not observe any role of IL-7 in the maintenance of these cells.

Using bone marrow chimeric mice, we found that IL-15 production by non-hematopoietic cells plays a key role for the maintenance of inflationary T cells in the lung. Our attempts to identify a specific non-hematopoietic cells type being responsible for IL-15 provision, using cell-type-specific IL-15-deficient hosts, remained largely unsuccessful, suggesting that we have either not targeted the right cell types or that there is considerably redundancy of IL-15 production by non-hematopoietic cells in the lung—a notion also supported by our observation of comparable IL-15 production in epithelial cells, blood and lymphatic endothelial cells. One of the stromal cells possibly involved in IL-15 provision are CCL19-producing FRCs, as we observed a trend for reduced survival of inflationary CD8 T cells in hosts deficient of IL-15 production in CCL19-expressing stromal cells. In contrast to a study that suggested pulmonary dendritic cells being an important IL-15 source for maintenance of effector CD8 T cells in the context of an influenza A infection, we did not identify DCs as an important cellular provider of IL-15 [42]. We propose that homeostatic IL-15 expression in non-hematopoietic cells represents a mechanism of inflationary CD8 T cell survival as we did not observe differences in their survival after transfer into naïve or latently MCMV infected hosts. Furthermore, our data suggest that IL-15 signals by non-hematopoietic cells are delivered via trans-presentation of the cytokine to the inflationary CD8 T cells, as adoptive transfer to IL-15Rα-deficient host pheno-copied the results of transfer into IL-15-deficient hosts.

Overall, we demonstrate that inflationary CMV-specific CD8 T cells residing in the lung tissue have a half-life of about 10–12 weeks, when external supply is abrogated, indicating that there is substantial need of continuous replenishment to maintain constant numbers as observed in latently CMV infected hosts. Their relatively long half-life is promoted solely by IL-15, expressed predominantly by non-hematopoietic cells in lung tissue, independent of CMV infection. The survival signals provided by IL-15 are co-dependent on IL-15Rα expression, suggesting that IL-15 acts via trans-presentation. Thus, CMV-driven inflationary CD8 T cell responses constitute a unique unit of "tissue-resident" T cells that is regulated and fuelled differently compared to T_RM_ CD8 T cells elicited upon vaccination or acute infection, offering interesting opportunities for CMV-based vaccine vectors.

## Material and methods

### Ethics statement

This study was conducted in accordance to the guidelines of the animal experimentation law (SR 455.163; TVV) of the Swiss Federal Government. The protocol was approved by Cantonal Veterinary Office of the canton Zurich, Switzerland (Permit number 127/2011, 146/2014).

### Mice

Wild-type C57BL/6J were purchased from Janvier Elevage (Le Genest Saint Isle, France). C57BL/6J, C57BL/6N-*Tg(TcraM38*,*TcrbM38)329Biat* (Maxi) [25], C57BL/6-*Il15ratm2*.*1Ama/J* (IL-15Rα^*fl/fl*^) [40], B6.*Tg(CMV-cre)1Cgn1/J* (*CMV*-Cre) [61], B6-(*Tg(Cdh5-cre)7Mlia/J*) (*VE-Cadherin*-Cre) [62], B6.Cg-*Tg(Itgax-cre)1-1Reiz/J* (CD11c-Cre) [63], C57BL/6N-*Tg(Ccl19-Cre)489Biat* (*Ccl19*-Cre) [64], C57BL/6NTac-*IL15tm1lmx* (IL-15^-/-^) [65], B6.*Cg-Gpi1a*.*Thy1a*.*Igha/J* (Thy1.1) [66] mice were housed and bred in specific pathogen-free facilities at the Eidgenössische Technische Hochschule (ETH) Hönggerberg. IL-15^*fl/fl*^ mice containing *LoxP* sites in the *Il15* gene in exon 5 were provided by Dr. K. Ikuta. Maxi transgenic (Ly5.1^+^) express a TCR specific for the MCMV peptide M38_316-323_ [25]. P14 transgenic (Ly5.1^+^) mice express a TCR specific for the LCMV peptide gp_33-41_ [34]. All mice were used 6–12 weeks of age and sex-matched within all experiments.

### Bone-marrow chimeras

Chimeric mice were generated by transferring 2–5 x 10^6^ bone marrow cells derived from Thy1.1 C57BL/6J or IL-15^-/-^ mice after lethal irradiation (950 rad) of recipient mice (IL-15^-/-^, Thy1.1 or C57BL/6J mice). Mice were treated in the drinking water with antibacterial Borgal 24% (Intervet, Boxmeer, Netherlands) during the first two weeks after reconstitution. Chimeric mice were reconstituted for 8–12 weeks before experiments.

### Viruses, infections and depletions

Recombinant MCMV lacking m157 (MCMVΔm157) was previously described and is referred to as MCMV in this study [67]. Recombinant MCMV expressing gp_33-41_ within the *M45* gene (referred to as MCMV-*M45*-gp33) or within the *ie2* gene (referred to as MCMV-*ie2*-gp33) was provided by Dr. L. Cicin-Sain. MCMV strains were propagated on MEFs [68] or M2-10B4 cells [69] as previously described. Virus titres in organs were determined by standard plaque-forming assays on M2-10B4 cells as previously described [69]. Infections were performed intravenously with 10^6^ pfu MCMV or 10^5^ pfu MCMV-*ie2*-gp33 or MCMV-*M45*-gp33.

Mice were treated intraperitoneally with depleting / neutralizing antibodies every second day for four weeks. For cytokine neutralization / receptor blockade 500 μg anti-IL-7 (M25), 200 μg anti-IL-2 (JESG-1A12) and 200 μg anti-CD122 (TM-β1) were injected intraperitoneally. NK cell depletion was performed by i. p. injection of 300 μg anti-NK1.1 (PK136).

### Adoptive transfer experiments

CD8 T cells from naïve Maxi or P14 mice were purified from splenocytes using anti-CD8α MACS beads (Miltenyi Biotech, Bergisch Gladbach, Germany) according to the manufacturer's instructions. 10^4^ Maxi or P14 CD8 T cells were adoptively transferred into recipient mice one day prior to infection. Memory subsets were generated in C57BL/6J mice and isolated at least 60 days post infection. Maxi or P14 T_EM_ (CD62L^-^) from lungs were sorted from donor mice using a BD FACSAria Sorter. Sorted Maxi or P14 T_EM_ (number varying inter-experimentally between 0.5–1.0 x 10^5^ cells) were transferred into new hosts.

### Lymphocyte isolation, intravascular, surface and intracellular staining

Lymphocytes were isolated from spleen, lung, liver and lungs as described before [70]. Blood samples were obtained from the tail vein. For intravascular staining, 5 μg fluorophore-coupled anti-CD8α (53–6.7) antibodies were injected intravenously 3 minutes prior to euthanasia as described previously [33, 71]. Surface staining of cells was performed for 20 min at room temperature in PBS before 5 min treatment with ACK lysis buffer for 5 min at room temperature. *In vitro* rIL-15 stimulation (50 ng/ml) was performed for 6 hours for pSTAT5 levels or 36 hours for Bcl-2 levels at 37°C (adapted from [72, 73]). Intracellular staining of Ki-67 and Bcl-2 was performed by fixing and permeabilizing cells using anti-mouse/rat FoxP3 staining set (eBioscience) according to the manufacturer's instructions. After fixation with 4% paraformaldehyde and permeabilisation with 90% methanol, intracellular staining of pSTAT5 was performed for 45 min in 1 x permeabilisation/wash buffer from FoxP3 staining kit (eBioscience). Multiparametric flow cytometric analysis was performed using LSRII flow cytometer (BD Biosciences, Allschwil, Switzerland) and FACSDiva software. Data was analysed using FlowJo software (Tree Star, San Carlos, CA, USA).

### Immunofluorescence microscopy

For the preparations of the lungs for microscopy, lungs were fixed by infusion of 1% PFA, followed by 20% sucrose (both 20 minutes) and finally, optimum cutting temperature (O.C.T.) was infused in the lung lumen. The organs were imbedded in O.C.T. and snap frozen in liquid nitrogen. Cryosections of 10 μM were made and air dried. For the staining, slides were re-hydrated in PBS and blocked with 10% rat serum in PBS. Slides were stained with antibodies diluted in PBS containing 1% rat serum and mounted with Mowiol.

### Antibodies and tetramers

APC- or PE-conjugated MHC class I tetramers were generated as described before [74]. Recombinant murine IL-15 protein was purchased from eBioscience. Fluorophore-conjugated antibodies were purchased from BioLegend (Lucerna Chem AG, Luzern, Switzerland), eBiosciences (Thermo Fisher Scientific, MA, USA) or BD Biosciences (Allschwil, Switzerland). The following antibodies (clone) were used for Flow cytometry or fluorescence microscopy: anti-CD8α (53–6.7), anti-CD8β.2 (53–6.8), anti-CD45.1 (A20), anti-CD45.2 (104), anti-CD90.1 (Ox-7), anti-CD90.2 (30-H12), anti-CD62L (MEL-14), anti-CD44 (IM7), anti-KLRG-1 (2F1), anti-Ki-67(SolA15), anti-Bcl-2 (BCL/10C4), anti-CD127 (A7R34), anti-CD4 (RM4-5), anti-CD3ε (145-2-C11), anti-CD69 (H1.2F3), anti-CD103 (2E7), anti-NKp46 (29A1.4), anti-CD49b (DX5), anti-NK1.1 (PK136), anti-CD31 (MEC13.3), anti-Podoplanin (8.1.1), anti-EpCAM (G8.8), anti-pSTAT5 (pY694). Live/Dead Fixable near-IR (Life Technologies) dead cell stain was used to exclude dead cells.

### Quantitative real-time PCR

Lungs and spleens from naïve mice were isolated and single cell suspensions were prepared. Hematopoietic and non-hematopoietic cell populations were sorted based on CD45 expression. RNA isolation was performed using Trizol LS reagnet (Life Technologies) as described before. 100 ng RNA was reverse-transcribed using the RT2 HT First Strand cDNA kit (QiaGEN, Hombrechtikon, Switzerland) according to the manufacturer's instructions. Real-time PCR was performed using a Rotorgene 3000 instrument (Corbett Research, Eight Miles Plains, Australia) to assess SYBR green incorporation (FastStart Universal SYBR Green Master, Roche Diagnostics, Switzerland). The following primer pairs were used for qPCR: IL-15: 5′-CATCCATCTCGTGCTACTTGTG-3′ *and* 5′-GCCTCTGTTTTAGGGAGACCT-3′ [75], β-actin: 5'-CCCTGAAGTACCCCATTGAAC-3' and 5'-CTTTTCACGGTTGGCCTTAG-3'. Data were analysed with Rotor-Gene 6000 Series Software (Qiagen, Hombrechtikon, Switzerland). Expression of the housekeeping gene β-acting was used as an internal control for normalisation. Relative expression levels were calculated according to the 2^-ΔΔCT^ method [76, 77].

### Statistical analysis

Statistical significance was determined as indicated by either non-parametric Mann-Whitney *U* test or unpaired two-tailed Student's *t* test using GraphPad Prism (La Jolla, CA, USA).

## Supporting information

S1 Fig Transgenic Maxi CD8 T cells do not exhibit a tissue-resident memory T cell phenotype. (A) Representative contour plot of CD69 and CD103 expression of Maxi cells in the lung and mean percentages of T_RM_ Maxi cells are shown as mean + SEM of n = 3 mice representative of two independent experiments. (B) Percentages of CD44^+^ CD62L^-^, CD69^+^ CD103^-^, KLRG1^-^ CD127^+^ and KLRG1^+^ CD127^-^ of Maxi cells in the lung (upper row) and spleen (lower row) at 6 months post infection are shown as mean + SEM of n = 3 mice representative of two independent experiments. (A, B) ns, not significant; *p<0.05;**p<0.01;***p<0.001. Statistical analyses were performed using the unpaired two-tailed Student's *t* test.
(TIF)

S2 Fig Half-life of lung-derived inflationary Maxi CD8 T cells in the spleen. Experimental setup: Naïve Maxi CD8 T cells were adoptively transferred into naïve C57BL/6 mice followed by i. v. 5 x 10^6^ pfu MCMVΔm157 infection. Effector-memory Maxi CD8 T cells were sorted from the lungs and transferred into infection-matched recipients. Total numbers of Maxi cells were assessed in the spleen at <1 and 12 weeks post transfer. Percentage transgenic Maxi cells recovered from the spleen are shown normalized to the total numbers recovered within the first week post transfer. Data are shown as mean + SEM of n = 6–8 mice pooled from two independent experiments.
(TIF)

S3 Fig Maintenance of effector-memory Maxi CD8 T cells is independent of NK cells. (A) Experimental setup: Naïve Maxi CD8 T cells were adoptively transferred into naïve C57BL/6 mice followed by i. v. MCMVΔm157 infection. Effector-memory Maxi T cells were sorted from the lungs and transferred into infection-matched C57BL/6 recipients. Recipients were administrated i. p. during 30 days with α-NK1.1 depleting antibody every second day. Total numbers of Maxi cells were assessed in the lungs at 4 weeks post transfer. (B) Total number of Maxi cells is shown as mean + SEM of n = 3–4 mice from one experiment. (C) Total numbers of NK cells in the lungs 30 days post transfer are shown as mean + SEM of n = 3–4 mice (D) Representative contour plots of NK cells in the two groups are shown. (B, C) ns, not significant; **p<0.01 Statistical analyses were performed using the unpaired two-tailed Student's *t* test.
(TIFF)

S4 Fig Chimerism of the bone-marrow chimeric mice. (A+B) CD4 T cells in the lung and spleen were analysed based on Thy1.1 and Thy1.2 expression and are shown as mean ± SEM of n = 4–6 mice representative from three independent experiments. (C) Representative flow cytometry contour plots are shown of CD4 T cells within the lung and spleen tissues of the chimeric mice.
(TIFF)

S5 Fig Several subsets of non-hematopoietic cells are able to express IL-15. (A) Lung tissues from naïve C57BL/6 mice were isolated and sorted into different subsets of stromal cells: Epithelial cells (CD45^-^ EpCAM^+^), blood endothelial cells (CD45^-^ EpCAM^-^ CD31^+^ Pdn^-^) and lymphatic endothelial cells (CD45^-^ EpCAM^-^ CD31^+^ Pdn^+^). The mRNA was isolated from all cell subsets and the relative expression levels were calculated using the ΔΔC_T_ method.
(TIFF)
