# Supplementary figures and images for: Tissue maintenance of CMV-specific inflationary memory T cells by IL-15
Source: PLoS Pathog. 2018 Apr 13;14(4):e1006993. doi: 10.1371/journal.ppat.1006993 (PMC5919076; doi:10.1371/journal.ppat.1006993)

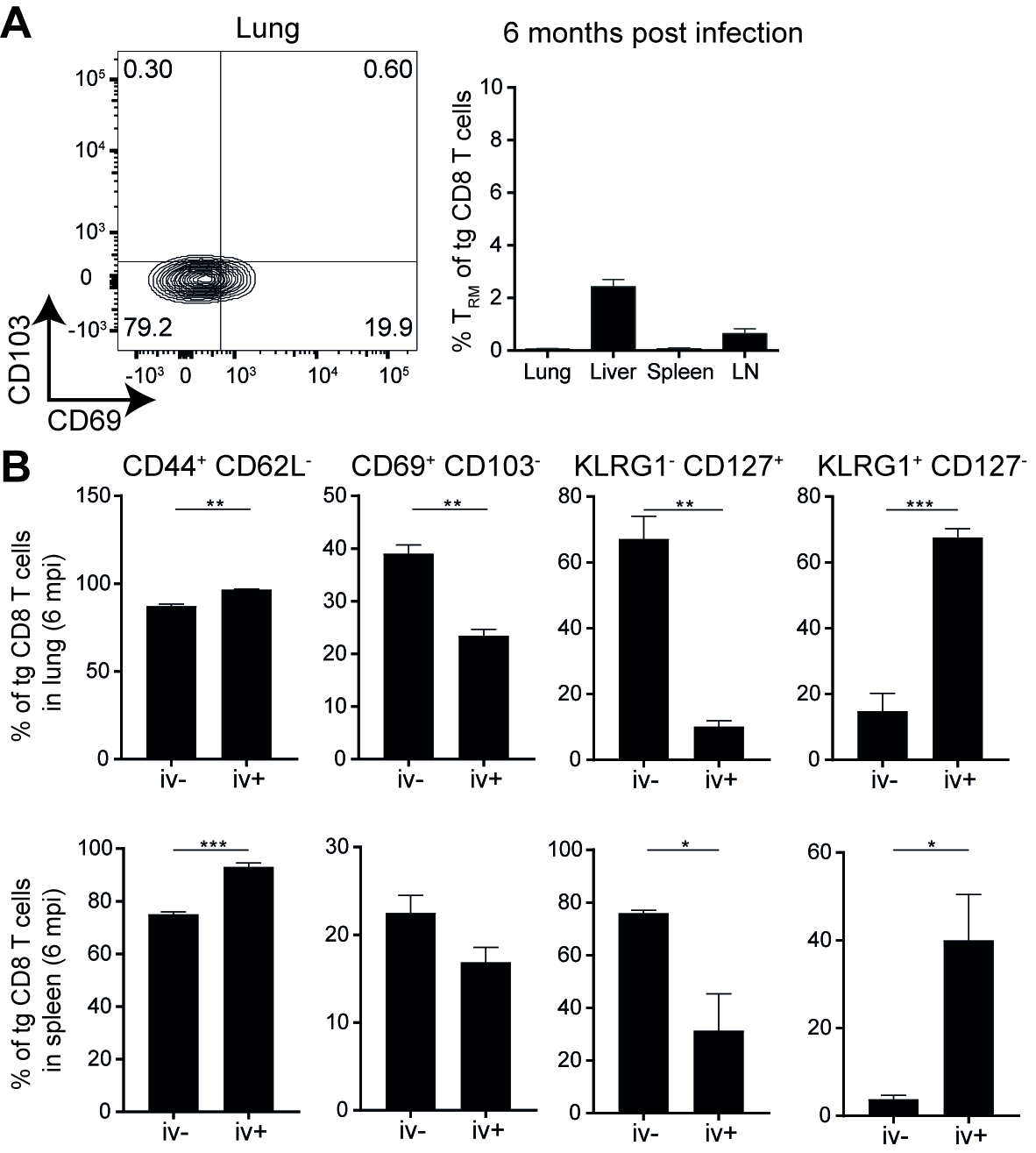

Supplement: S1 Fig — (A) Representative contour plot of CD69 and CD103 expression of Maxi cells in the lung and mean percentages of TRM Maxi cells are shown as mean + SEM of n = 3 mice representative of two independent experiments. (B) Percentages of CD44+ CD62L-, CD69+ CD103-, KLRG1- CD127+ and KLRG1+ CD127- of Maxi cells in the lung (upper row) and spleen (lower row) at 6 months post infection are shown as mean + SEM of n = 3 mice representative of two independent experiments. (A, B) ns, not significant; *p<0.05;**p<0.01;***p<0.001. Statistical analyses were performed using the unpaired two-tailed Student's t test. (TIF) [file ppat.1006993.s001.tif]

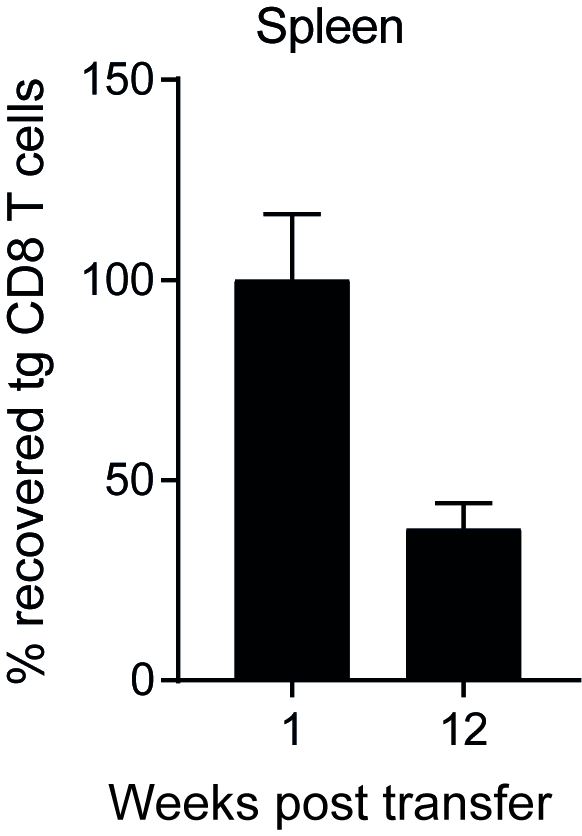

Supplement: S2 Fig — Experimental setup: Naïve Maxi CD8 T cells were adoptively transferred into naïve C57BL/6 mice followed by i. v. 5 x 10^6^ pfu MCMVΔm157 infection. Effector-memory Maxi CD8 T cells were sorted from the lungs and transferred into infection-matched recipients. Total numbers of Maxi cells were assessed in the spleen at <1 and 12 weeks post transfer. Percentage transgenic Maxi cells recovered from the spleen are shown normalized to the total numbers recovered within the first week post transfer. Data are shown as mean + SEM of n = 6–8 mice pooled from two independent experiments. (TIF) [file ppat.1006993.s002.tif]

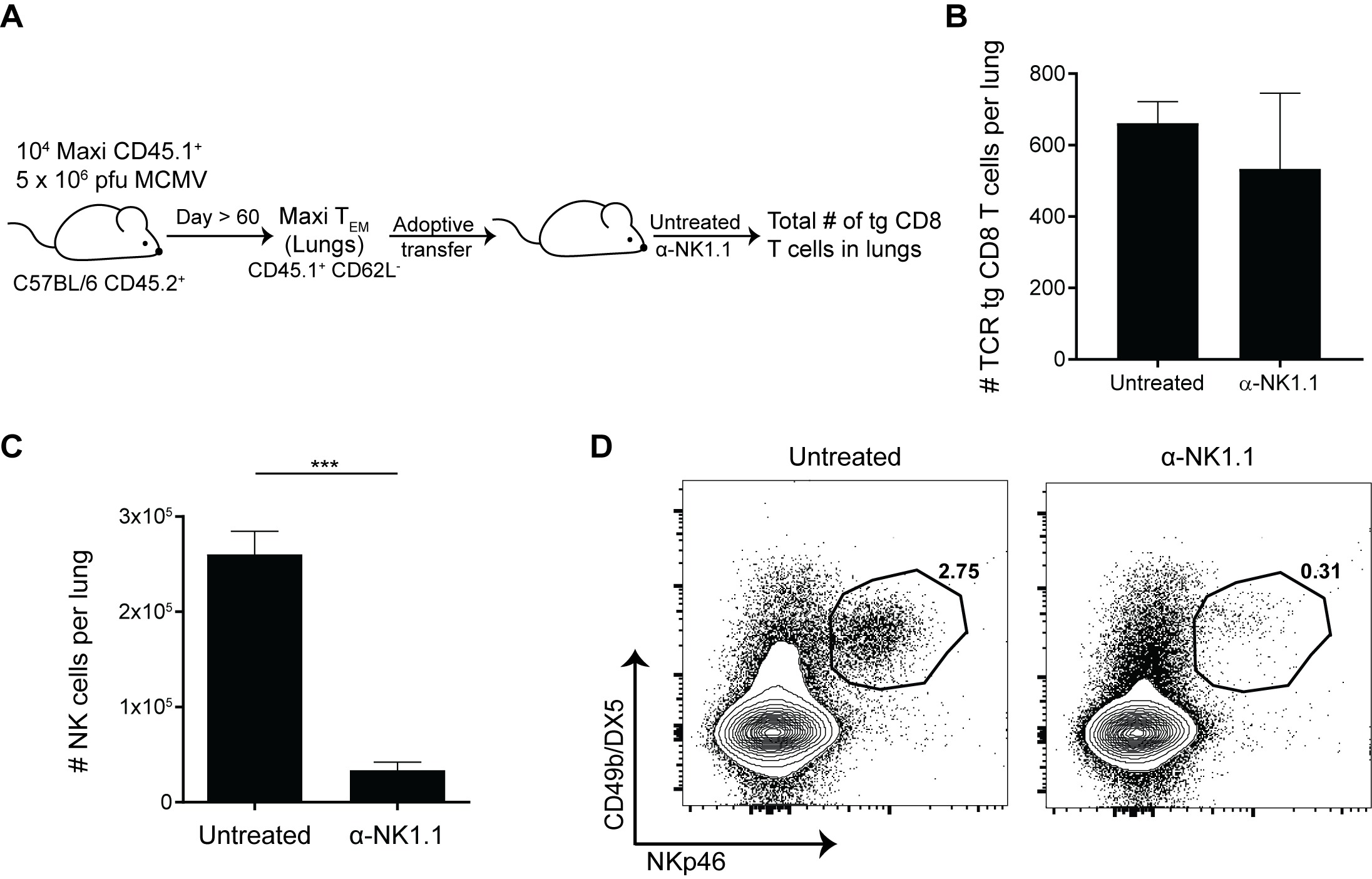

Supplement: S3 Fig — (A) Experimental setup: Naïve Maxi CD8 T cells were adoptively transferred into naïve C57BL/6 mice followed by i. v. MCMVΔm157 infection. Effector-memory Maxi T cells were sorted from the lungs and transferred into infection-matched C57BL/6 recipients. Recipients were administrated i. p. during 30 days with α-NK1.1 depleting antibody every second day. Total numbers of Maxi cells were assessed in the lungs at 4 weeks post transfer. (B) Total number of Maxi cells is shown as mean + SEM of n = 3–4 mice from one experiment. (C) Total numbers of NK cells in the lungs 30 days post transfer are shown as mean + SEM of n = 3–4 mice (D) Representative contour plots of NK cells in the two groups are shown. (B, C) ns, not significant; **p<0.01 Statistical analyses were performed using the unpaired two-tailed Student's t test. (TIFF) [file ppat.1006993.s003.tiff]

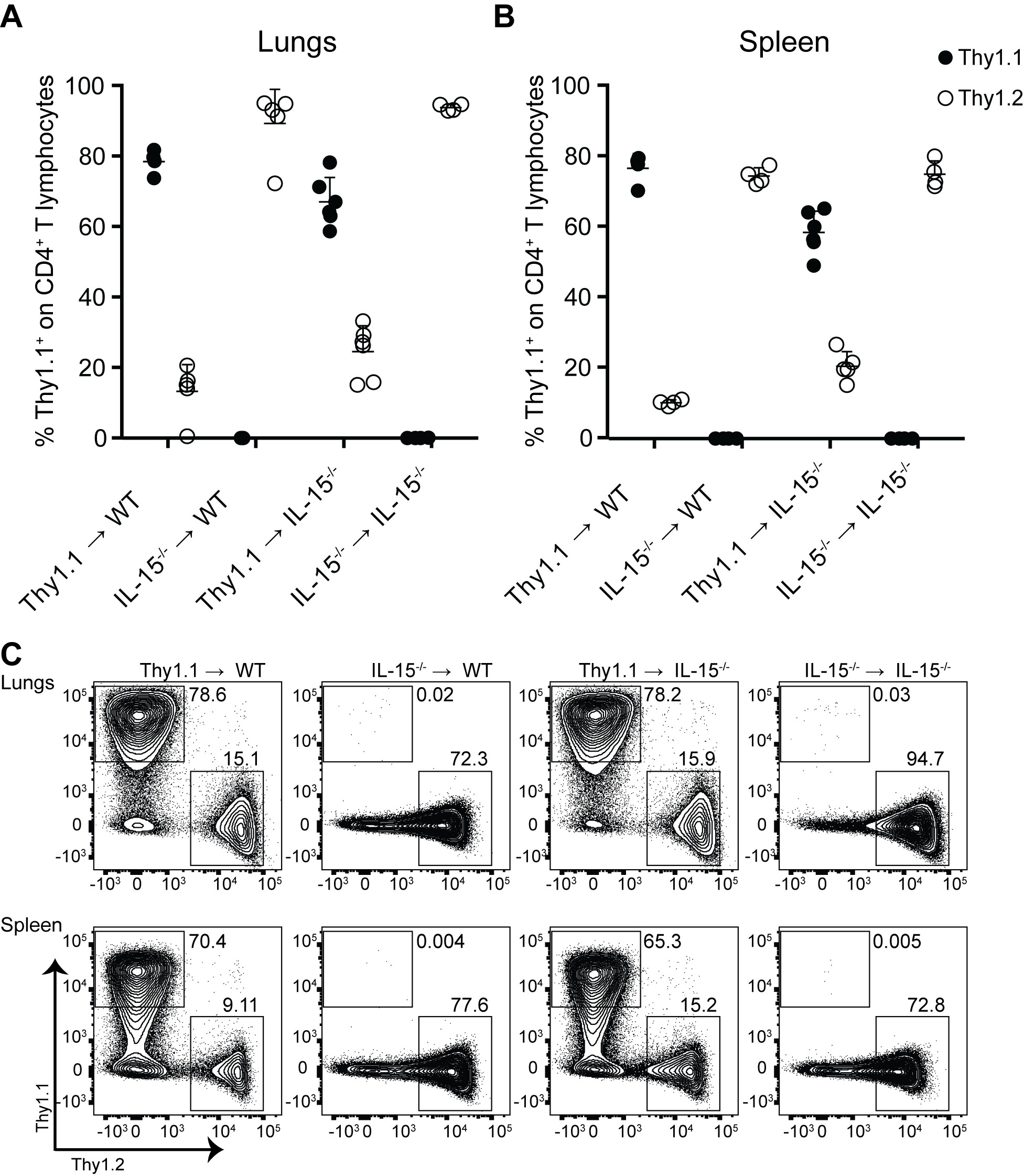

Supplement: S4 Fig — (A+B) CD4 T cells in the lung and spleen were analysed based on Thy1.1 and Thy1.2 expression and are shown as mean ± SEM of n = 4–6 mice representative from three independent experiments. (C) Representative flow cytometry contour plots are shown of CD4 T cells within the lung and spleen tissues of the chimeric mice. (TIFF) [file ppat.1006993.s004.tiff]

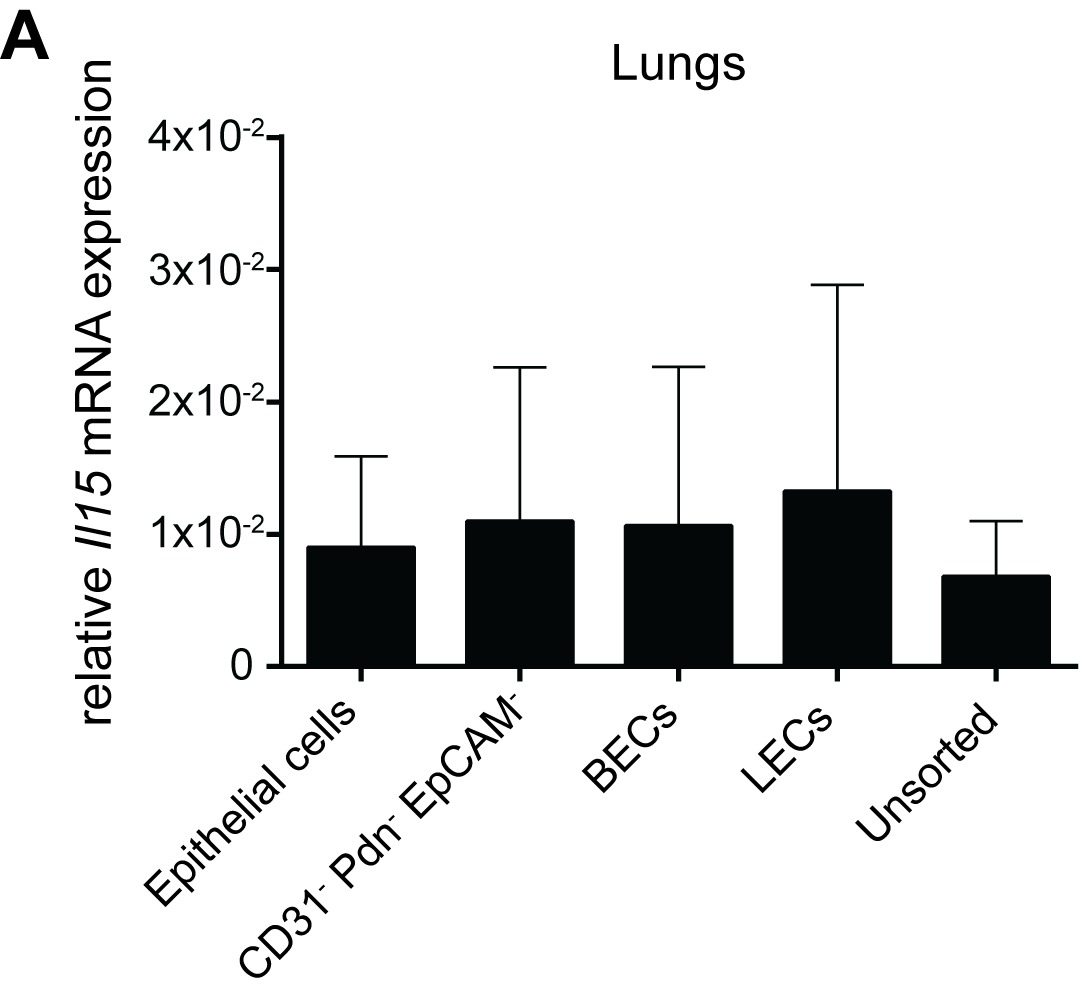

Supplement: S5 Fig — (A) Lung tissues from naïve C57BL/6 mice were isolated and sorted into different subsets of stromal cells: Epithelial cells (CD45- EpCAM+), blood endothelial cells (CD45- EpCAM- CD31+ Pdn-) and lymphatic endothelial cells (CD45- EpCAM- CD31+ Pdn+). The mRNA was isolated from all cell subsets and the relative expression levels were calculated using the ΔΔCT method. (TIFF) [file ppat.1006993.s005.tiff]
